# Combined Hyperglycemic Hyperosmolar Syndrome and Diabetic Ketoacidosis Associated with COVID-19 in a Pediatric Patient

**DOI:** 10.1155/2021/6429710

**Published:** 2021-11-27

**Authors:** Yu Shan Tseng, Bradley Tilford, Usha Sethuraman, Katherine Cashen

**Affiliations:** ^1^Department of Pediatrics, Division of Pediatric Critical Care, Children's Hospital of Michigan, Detroit, Michigan, USA; ^2^Department of Pediatrics, Division of Pediatric Critical Care, Central Michigan University/Children's Hospital of Michigan, Detroit, Michigan, USA; ^3^Department of Pediatrics, Division of Pediatric Emergency Medicine, Central Michigan University/Children's Hospital of Michigan, Detroit, Michigan, USA

## Abstract

Although most children with coronavirus disease 2019 (COVID-19) are asymptomatic or only with mild symptoms, many symptomatic children still require admission to the intensive care unit. Multiple cases of diabetic ketoacidosis (DKA) and hyperosmolar hyperglycemic syndrome (HHS) associated with COVID-19 have been reported in adults. However, to our knowledge, only few similar cases have been published in the pediatric population. We report one of the first few severe cases of mixed HHS with DKA associated with COVID-19 in an adolescent. Our patient was successfully treated with intravenous immunoglobulin, Remdesivir, and methylprednisolone. As the pandemic continues, clinicians should be aware of this syndrome and consider early use of Remdesivir and corticosteroids. Further studies are required to understand the pathophysiology of this syndrome occurring with COVID-19.

## 1. Introduction

The novel Severe Acute Respiratory Syndrome Coronavirus 2 (SARS-CoV-2) has rapidly spread throughout the world. Although the number of children with symptomatic coronavirus disease (COVID-19) is significantly less than adults, severe cases have been described [1–4]. Severe diabetic ketoacidosis (DKA) and hyperosmolar hyperglycemic syndrome (HHS) have been associated with COVID-19 infection in adults [5–7], and it has been reported that HHS mixed with DKA has higher mortality compared to isolated HHS or DKA alone [8]. We report one of the first few cases of combined DKA and HHS associated with active COVID-19 in a pediatric patient with multisystem inflammatory syndrome in children (MIS-C). Even pediatric patients without known diabetes can present with isolated or combined DKA/HHS as the first manifestation of COVID-19 infection.

## 2. Case Presentation

A 14-year-old obese (78 kg, 160 cm, and BMI of 30.5) African American female with no other significant past medical history presented to the emergency department (ED) with altered mental status. On the day of presentation, she woke up with abdominal pain. She was confused and vomited when she got up from the bed. There was no history of fever, cough, diarrhea, ingestion, or trauma. She was evaluated for this altered sensorium at an outside institution, where her vitals included temperature 36.8°C, heart rate 120/min, blood pressure 113/75 mmHg, and respiratory rate 34/min with oxygen saturation of 100% on room air. She was arousable but not oriented to time, place, and person and moaned to pain stimulation. Her physical exam was notable for dried mucous membrane and cold extremities with capillary refill time of 3 seconds. Laboratory results showed marked hyperglycemia of 1858 mg/dL, sodium of 121 mmol/L (corrected 149 mmol/L), blood urea nitrogen of 47 mg/dL, and creatinine of 4.5 mg/dL (Table 1). Her calculated osmolality was 430 mOsm/kg. Arterial pH was 6.9 with partial pressure of carbon dioxide of 20 mmHg and bicarbonate of 6 mEq/L. Beta-hydroxybutyrate was 46 mg/dL. She was leukopenic (2.8 K/cumm) with mild lymphopenia (0.7 K/cumm) and thrombocytopenia (0.7 K/cumm) and elevated lipase (666 U/L). She had elevated troponin (343 ng/L (reference 3–17 ng/L)), C reactive protein (210 U/L), and d-dimer (4.15 mg/L.) In addition, she had rhabdomyolysis with creatinine phosphokinase peak of 11,872 U/L. COVID-19 PCR was positive. Urine and serum drug screens were negative. Head Computed Tomography (CT) was read as normal. She was diagnosed with type 2 diabetes as her autoantibodies (islet cells, GAD-65, IA-2, and insulin antibodies) were all negative.

Initial resuscitation involved boluses of a total of 3 liters of isotonic fluid. The patient was started on our institutional DKA protocol with 5% fluid deficit replacement in addition to her maintenance requirement over 24 hrs and insulin drip at 0.05 U/kg/hr. She developed fever and vasodilatory shock with hypotension and required additional fluid resuscitation and initiation of inotropic support with epinephrine, vasopressin, and norepinephrine and broad-spectrum antibiotics. In addition, her fluid replacement rate was increased to cover a 12% fluid deficit over 24 hrs in addition to her maintenance requirement to match the higher percentage of dehydration in HHS.

**Figure 1:**
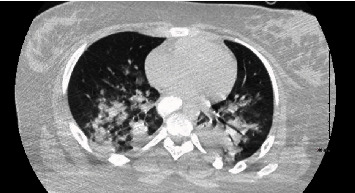
CT of the lungs demonstrates bilateral mid- and lower lung patchy consolidations and ground-glass opacities extending into the bilateral lung bases. Bilateral air bronchograms are noted.

**Figure 2:**
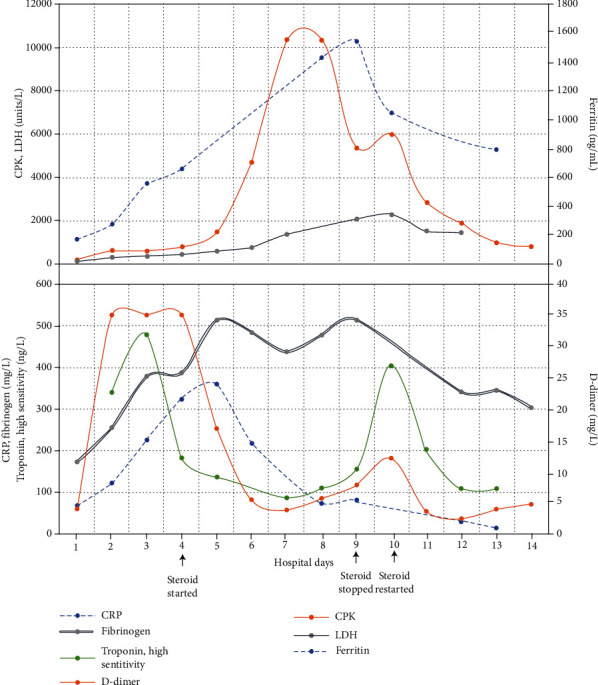
Inflammatory markers over time.

She was transferred to our pediatric intensive care unit (PICU) where she was intubated and mechanically ventilated due to acute respiratory distress syndrome and multiorgan failure. She was febrile with persistent hypotension requiring escalation of inotropes. Chest CT showed patchy consolidation and ground-glass opacities (Figure 1), and abdominal CT showed a small amount of free fluid in the pelvis but no intra-abdominal abscess or source of infection. She met the case definition of MIS-C due to fever, laboratory evidence of inflammation with more than 2 organ system failures, and COVID-19 positive reverse transcriptase polymerase chain reaction (RT PCR). She was treated with Remdesivir (per compassionate use guidelines and administered according to manufacturer recommendations after obtaining parental consent), intravenous immunoglobulin 1 g/kg (IVIG), and methylprednisolone (40 mg IV twice a day for 3 days) for acute respiratory distress syndrome and ongoing systemic inflammation. When her methylprednisolone was discontinued, her inflammatory markers, fever, and renal function worsened, so dosing was increased to 200 mg twice daily and weaned slowly over a month (Figure 2). Echocardiography showed normalization of the septal dyskinesia and normal left and right ventricular function. With these interventions, she clinically improved and was extubated 8 days after admission.

As the patient's HHS slowly improved, so did her mental status. Despite her clinical improvement, she remained febrile and her inflammatory markers continued to remain elevated with elevated arterial lactate around 2-3 gm/dL. With these signs of continued systemic inflammation, her methylprednisolone dose was increased as described above. She had a good response and defervesced and had improvement in her inflammatory markers and resolution of lactate acidosis. She was transferred to the general acute pediatric floor after 2 weeks in the PICU and later to the inpatient rehabilitation unit on subcutaneous insulin. Her HHS resolved, renal function and mental status improved, and ultimately recovered to her preillness baseline. She was discharged home after 2 weeks in inpatient rehabilitation. Her MRI at discharge showed cerebellar hemisphere and vermis volume loss and subtle supratentorial volume loss.

## 3. Discussion

Hyperglycemic hyperosmolar syndrome (HHS), defined as serum glucose > 600 mg/dL, serum osmolarity > 330 mOsm, and mild ketosis, is a distinct diagnosis from diabetes ketoacidosis (DKA) but can occur concomitantly with DKA. Over the last twenty years, there have seen an increased number of HHS reported in adolescents [9]. A review article reported 71 pediatric HHS cases from 2001 to 2008 with a mortality rate of 37% [10]. However, severe HHS with glucose > 1000 mixed with diabetic ketoacidosis is still a rarity in the pediatric population.

Diabetes is a known predisposing factor for severe COVID-19 infection [11]. There have been emerging reports of adult patients with diabetes admitted for combined DKA and HHS with positive COVID-19 with no other predisposing triggers for their hyperglycemic crises [5, 6, 12]. To our knowledge, our patient is one of the first reports of a child with MIS-C secondary to COVID-19 with severe mixed HHS and DKA with encephalopathy who survived to discharge. Singh et al. published a case series of 11 cases of combined DKA and HHS in COVID-19 patients. The case series included an overweight 12-year-old male with undiagnosed diabetes mellitus. The patient presented with a pH of 6.81, blood glucose of 1385 mg/dL, and effective osmolarity of 357 mOsm/kg. Unlike our patient, the child did not progress to respiratory failure or multiple organ dysfunction syndrome. He recovered well and was discharged home [13]. Chan et al. reported the case of a 19-year-old male with morbid obesity who was admitted with combined DKA and HHS, with an initial blood glucose of 1112 mg/dL and osmolarity of 307 mOsm/kg. He went on to develop multiorgan failure, including renal failure requiring continuous renal replacement therapy, hypoxic respiratory failure, and persistent atrial flutter. Unfortunately, he died after 13 days of hospitalization [12].

The case definition of MIS-C includes individuals aged <21 years presenting with fever, laboratory evidence of systemic inflammation, and multisystem organ involvement and tested positive for current or recent SARS-CoV-2 infection [14, 15]. Although we have treated several known diabetic pediatric patients with MIS-C at our institution, those patients have not had the degree of hyperglycemia as the patient described in this case report.

DKA and HHS in patients with new-onset and established diabetes are often precipitated by infection and stress. However, we speculate that there may be a potentiated diabetogenic effect from COVID-19 that provokes certain patients into hyperglycemic crises. Though the exact pathophysiology of hyperglycemic crises in patients with COVID-19 is still unclear, there are a few hypothesized mechanisms. The angiotensin-converting enzyme 2 (ACE2) receptor is expressed in the pancreas (pancreatic ductal, acinar, and islet cells), as well as the lungs, kidneys, and the gastrointestinal system [16]. Both COVID-19 and SARS coronavirus (SARS-CoV-1) have been shown to bind to and downregulate ACE2 receptors [17, 18]. This could lead to increased concentration of angiotensin II, which could delay insulin secretion and decrease blood flow to the pancreatic islet cells, both resulting in hyperglycemia [19]. Another proposed mechanism is that COVID-19 and SARS-CoV-1 may directly cause cellular damage to the pancreatic islet cells, particularly beta cells, resulting in decreased insulin secretion [20].

The endocrine and exocrine pancreas are closely related, both anatomically and functionally. Pancreatitis is a known complication of DKA/HHS, and conversely, acute pancreatitis can incite DKA/HHS. The relationship is bidirectional. However, when there is a very high plasma glycerol level from rapid breakdowns of triglycerides, such as in DKA, false-positive elevation of amylase and lipase can happen [21–23]. Our patient had elevated lipase, but her contrast-enhanced abdominal CT was negative for pancreatic edema or enhancement. Therefore, we could not confirm the diagnosis of pancreatitis. Furthermore, COVID-19 is also associated with acute pancreatitis. Although the clear pathogenesis is still unknown, it is thought to be from the direct cytotoxic effect of the virus on the exocrine pancreas, secondary to cytokine storm, or damage from microvascular thrombosis [24–26].

In our institution, we have seen twice the number of patients with severe HHS mixed with DKA (serum glucose from 1000 mg/dL to as high as 2100 mg/dL and osmolality > 400 mOsm/kg) in 2021 compared to 2019. Unfortunately, since early in the pandemic, COVID-19 PCR and serology were not readily available; we do not know the COVID-19 status of all the pediatric patients with severe HHS admitted to our PICU since the beginning of the pandemic. In addition to the pathophysiologic explanation for increased HHS in COVID-19, another contributing factor may be related to patients delaying seeking medical care in fear of contracting COVID-19. The signs and symptoms of HHS are usually insidious compared to DKA, and patients are more likely to be symptomatic for days to weeks before they present to the ED; this may be another explanation for the severity of intravascular depletion and metabolic derangements in our patient.

Of note, our patient developed rhabdomyolysis during her hospitalization, which is a known complication of HHS from hypophosphatemia. There are multiple reported cases of COVID-19-associated rhabdomyolysis both in adults and children [27–29]. Chen et al. reported that muscle biopsies in SARS-CoV-1 patients with rhabdomyolysis found no viral particles on pathology and postulated that SARS-CoV-associated rhabdomyolysis may have been secondary to cytokine storm [30]. This could be extrapolated to COVID-19-associated rhabdomyolysis. Other important considerations in the care of our patient involved balancing the multiorgan failure with the need for adequate fluid hydration. This child developed acute respiratory distress syndrome yet needed adequate intravascular volume for HHS and rhabdomyolysis. This case highlights the complexity in diagnosis, initial resuscitation, and ongoing treatment strategy for a novel presentation of HHS in acute COVID-19 infection with MIS-C.

## 4. Conclusion

Herein, we report one of the first few pediatric cases of life-threatening HHS with DKA in the setting of acute COVID-19 infection. Our patient developed multiorgan failure and met the case definition for MIS-C. She was successfully treated with IVIG, Remdesivir, and methylprednisolone. This case highlights a novel presentation of a new infection, and studies focused on the epidemiology and pathogenesis of COVID-19 on glucose dysregulation in the pediatric population are needed. It is crucial to recognize that pediatric patients with or without diabetes can present with isolated or combined DKA/HHS as the first manifestation of COVID-19 infection.

## Figures and Tables

**Table 1:** Initial laboratory values and peaked values for the inflammatory markers.

Variable	Initial values	Reference ranges
HbA1c (%)	13	4–6
pH	6.9	7.36–7.44
Bicarbonate (mEq/L)	6	21–31
Glucose (mg/dL)	1858	70–110
Beta-hydroxybutyrate (mg/dL)	46	0.2–2.8
Anion gap (mEq/L)	22	3–10
Effective osmolarity (mOsm/kg)	345	283–299
WBC (K/mm^3^)	9.7	4.5–11
Hemoglobin (g/dL)	15.7	12–16
Platelets (K/mm^3^)	202	140–440
Sodium (mEq/L)	121	135–145
Potassium (mEq/L)	6.1	3.5–5
Chloride (mEq/L)	93	98-107
Phosphorous (mg/dL)	<1	2.5–5
BUN (mg/dL)	47	7–23
Creatinine (mg/dL)	4.5	0.6–1.3
Amylase (U/L)	120	40–140
Lipase (U/L)	666	0–160
Troponin (ng/L)	343 (peak: 481)	3–17
CRP (mg/L)	210 (peak: 362)	<10
Ferritin (ng/mL)	172 (peak: 1546)	12–300
LDH (U/L)	120 (peak: 2295)	140–271
D-dimer (*μ*g/mL)	4.15 (peak: 12.26)	<0.5
Fibrinogen (mg/dL)	175 (peak: 516)	183–503
CPK (U/L)	210 (peak: 10375)	20-200
